# SIADH Induced by Pharyngeal Squamous Cell Carcinoma: Case Report and Literature Review

**DOI:** 10.1155/2016/3186714

**Published:** 2016-08-22

**Authors:** Hafiz Muhammad Sharjeel Arshad, Aleida Rodriguez, Faten Suhail

**Affiliations:** Department of Internal Medicine, University of Illinois at Chicago/Advocate Christ Medical Center, Oak Lawn, IL 60453, USA

## Abstract

*Background*. The Syndrome of Inappropriate Antidiuretic Hormone (SIADH) is considered to be the most common cause of euvolemic hyponatremia. The most common malignancy associated with SIADH is small cell lung cancer. We present a rare case of a patient with SIADH secondary to well differentiated squamous cell carcinoma of the naso-oropharynx.* Case*. A 46-year-old Caucasian woman presented to emergency department with four-week history of progressive dysphagia. On examination, she was found to have a pharyngeal mass. CT scan and MRI of neck confirmed a mass highly suspicious of carcinoma. Patient's serum sodium level decreased to 118 mEq/L and other labs including serum and urine osmolality confirmed SIADH. She was started on fluid restriction and oral sodium tablets which gradually improved her serum sodium levels. Biopsy confirmed diagnosis of squamous cell carcinoma of pharynx.* Conclusion*. SIADH can be caused by squamous cell carcinoma. Appropriate management includes fluid restriction.

## 1. Introduction

The Syndrome of Inappropriate Antidiuretic Hormone (SIADH) is considered to be the most common cause of euvolemic hyponatremia. Common causes of SIADH include pulmonary disease such as pneumonia, tuberculosis, pleural effusions, and CNS disorders such as subarachnoid hemorrhage and meningitis. SIADH is also associated with malignancy. The most common malignancy associated with SIADH is small cell lung cancer, accounting for 70% of cases. We present a rare case of a patient with SIADH secondary to well differentiated squamous cell carcinoma of the naso-oropharynx.

## 2. Case

A 46-year-old Caucasian woman with past medical history significant for tobacco and alcohol abuse presented to emergency department with four-week history of progressive dysphagia. One week priorly, she had presented with a complaint of sore throat and was diagnosed with streptococcal pharyngitis for which she was given course of antibiotics in another hospital.

Her symptoms persisted and she presented with progressively worsening sore throat, odynophagia, headache, and unintentional weight loss of approximately 15 pounds in 2 months. Headache was generalized, mild-moderate in intensity, and associated with nausea but no vomiting. Her physical examination was remarkable for cachexia with poor oral hygiene, oral thrush, erythematous pharynx with exudates, and foul odor. An erythematous mass in the left oropharynx and deviation of the uvula towards right were observed. Tender submandibular and cervical lymphadenopathy was also found on physical examination.

**Figure 1:**
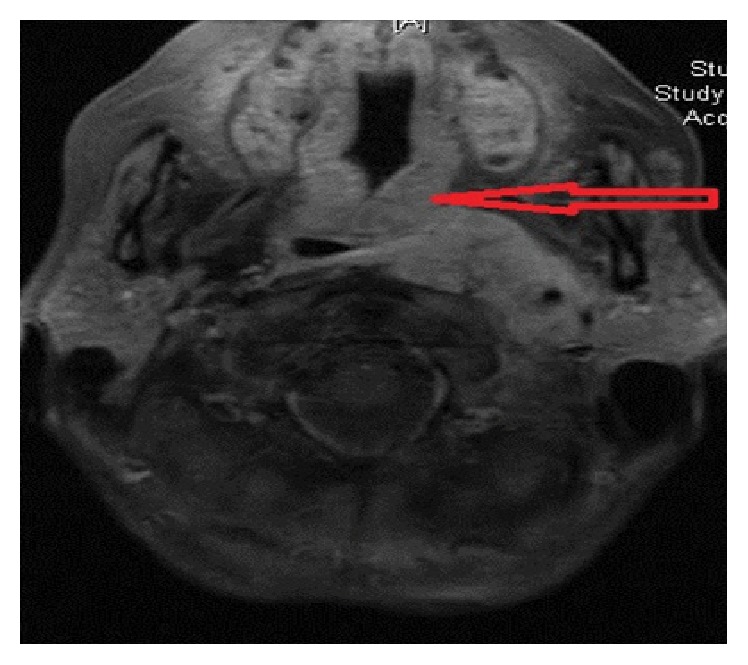
MRI of neck showing pharyngeal mass.

Laboratory evaluation on initial presentation showed a sodium level of 118 meq/L, blood urea nitrogen of 4 mg/dL, and creatinine of 0.56 mg/dL. A CT scan of neck without contrast (due to allergy to IV contrast) was performed which showed a density at the level of the left posterior nasopharynx and oropharynx extending into the left parapharyngeal space. An MRI of neck without contrast also showed same mass (see Figure 1).

**Figure 2:**
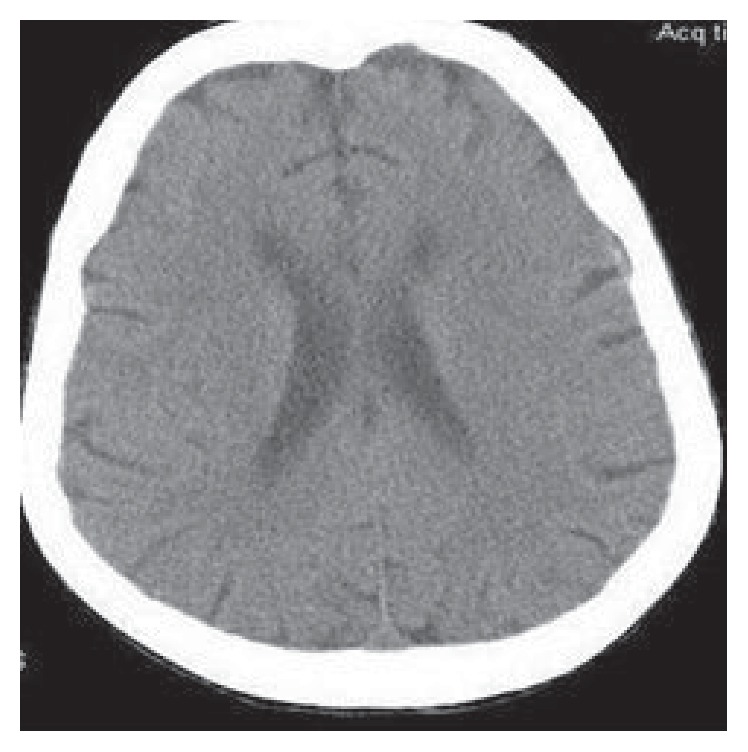
CT scan of head without any intracranial lesion.

Patient was only taking tylenol as needed at the time of admission. Based on history and physical examination, her hyponatremia was suspected secondary to hypovolemia and malnutrition. She was started on normal saline at a rate of 125 mL/hour which improved her sodium level to 132 meq/L over next three days. Patient received tylenol, vancomycin, aztreonam, dexamethasone, zofran, and lovenox during this time. On day four, her serum sodium dropped to 115 meq/L. Patient, once again, remained asymptomatic. At this point, further workup of the etiology of hyponatremia was started with a high suspicion of SIADH and IV fluids were stopped. Plasma osmolality was 239 osmol/L, urine osmolality was 361 osmol/L, urine sodium was >40 meq/L, and serum uric acid was 1.9 mg/dL. TSH and cortisol levels were within normal limits. A CT scan of head was performed which did not find any intracranial lesions (see Figure 2). X-ray of chest did not show evidence of any pulmonary disease.

**Figure 3:**
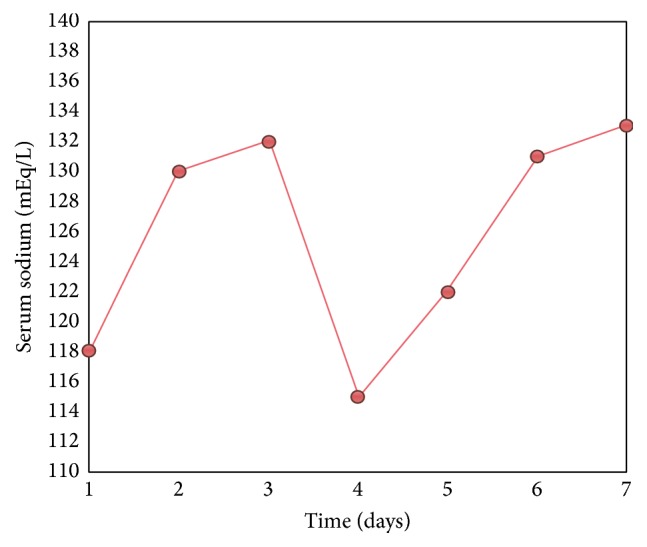
Changes in serum sodium levels during hospital stay.

Based on above mentioned workup, diagnosis of SIADH was made and patient was put on fluid restriction (<800 mL/day) and given 2 doses of 20 mg IV furosemide. Sodium chloride 100 mg BID tablets were also started. Over the course of next seven days, patient's sodium gradually improved and became normal after fourteen days (see Figure 3).

**Figure 4:**
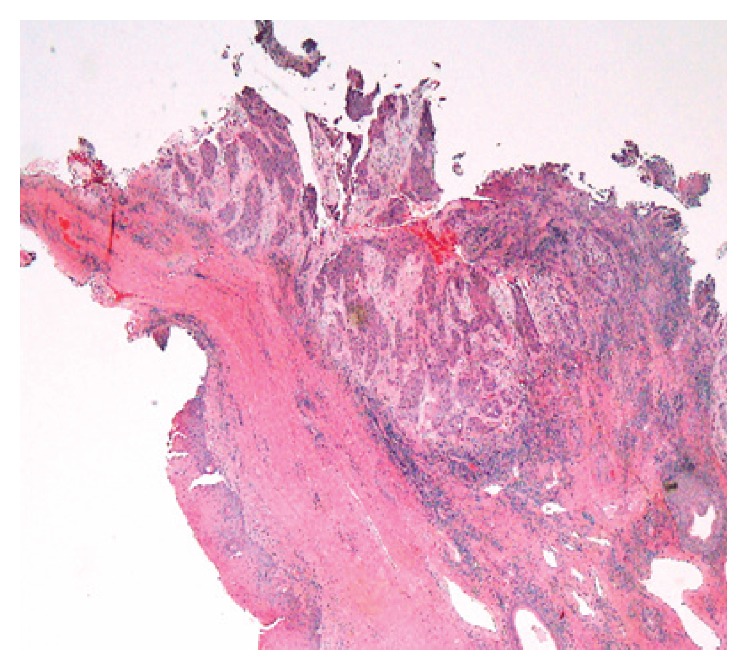
Low power view showing tumor on mucosal surface, invading in submucosa.

A biopsy of neck mass was performed which showed well differentiated squamous cell carcinoma. The underlying etiology of her SIADH was attributed to biopsy-proven well differentiated squamous cell carcinoma of the oronasopharynx (see Figure 4).

## 3. Discussion

SIADH is one of the most common causes of euvolemic hyponatremia. SIADH is often associated with cancer. Approximately 67% of SIADH cases are reported to be caused by cancer. The majority of these cases (70%) have been linked to small cell carcinoma of the lung [1]. Head and neck cancers are linked with 1.5% of SIADH cases [2].

 Schwartz et al. first described SIADH in two patients with bronchogenic small cell carcinoma in 1957 [3]. They suggested that small cell carcinoma produced some quantity of antidiuretic hormone. This hormone was later on described as arginine vasopressin (AVP) by Bleich. Bleich and Boro also discovered the AVP-regulated water channels in the kidneys which were later on called “aquaporins” [4]. Schwartz's hypothesis was proven by detecting ectopic AVP in small cell carcinoma of the lung with inappropriate diuresis [5].

In 1976, Moses et al. first described SIADH in patients with squamous cell carcinoma [6]. However, they were unable to explain the mechanism behind it. Although researchers have found AVP gene in small cell and undifferentiated carcinoma, they have failed to find AVP gene in squamous cell carcinoma [7]. In 2004, Lee proposed that neuropeptide Y is increased in squamous cell carcinoma patients which in turn increases endogenous AVP production leading to SIADH [8]. In contrast, small cell carcinoma produces AVP directly. Carotid manipulation after surgical resection of malignancy leading to stimulation of baroreceptors associated with carotid bulb is another proposed phenomenon [9, 10]. SIADH has also been associated with chemotherapy and radiotherapy. Direct effect of chemotherapeutic medications and obstruction of cerebral blood flow induced by radiotherapy leading to increased AVP production have been proposed as possible mechanisms [11–13].

Clinical features of SIADH induced by squamous cell carcinoma can range from mild symptoms like fatigue, anorexia, and lethargy to severe neurological manifestations like convulsions and coma. However, most patients are asymptomatic [14].

Kettler et al. published a review of 29 patients with hyponatremia associated with oropharyngeal carcinoma. In 19 patients, the hyponatremia was attributable to a decreased effective arterial blood volume. In 10 patients, presence of SIADH leading to hyponatremia was described. The etiologies of hyponatremia (SIADH or non-SIADH) were best differentiated by the plasma urea concentration (less than 30 mg/dL in SIADH), urate concentration (less than 4,0 mg/dL in SIADH), and creatinine concentration (less than 0,9 mg/dL in SIADH) [15].

A brief review of cases of SIADH induced by pharyngeal squamous cell carcinoma in a chronological order is shown in Table 1.

Management of inpatient hyponatremia requires accurate diagnosis of underlying etiology as treatment is dependent on the cause. Grant et al. recently published algorithm for inpatient management of hyponatremia [16]. Symptomatic hyponatremia requires immediate hypertonic saline (3%). In asymptomatic patients, management depends on patient's volume status. SIADH is generally classified as euvolemic hyponatremia. If history, physical examination, and labs (serum osmolality, urine osmolality, and urine sodium excretion) indicate SIADH as most probable etiology for hyponatremia, fluid restriction (500–1000 mL daily depending on severity of hyponatremia) is the first step in treatment [17]. Serum sodium levels need to be monitored every six hours initially to monitor appropriate response (<8–10 meq/24 hours increase in serum sodium) to avoid osmotic demyelination syndrome. Oral salt tablets have also been used to correct serum sodium levels in SIADH. Salt tablets tend to raise serum osmolality, causing excessive loss of salt in urine leading to dieresis. This diuresis eventually raises serum sodium levels. Pharmacological therapy for SIADH includes use of demeclocycline, vaptans, and furosemide. Demeclocycline is a tetracycline derivative which induces nephrogenic diabetes insipidus, thus leading to increased serum sodium levels [18, 19]. Vasopressin-2 receptor antagonists tolvaptan and conivaptan have also been used very regularly recently for treatment of hyponatremia. Petereit et al. demonstrated that tolvaptan (15 mg/day) was effective in stabilization of serum sodium levels in patients with SIADH [20]. Furosemide is often used in combination with salt tablets. It decreases reabsorption of sodium in Loop of Henle, thus causing diuresis and eventually slowly increasing serum sodium levels. Decaux et al. confirmed utility of furosemide for management of SIADH [21].

In summary, this case illustrates the importance of the initial evaluation of hyponatremia and the need for effective data gathering in exploring the possible etiologies of hyponatremia. If patients are not responding to IV fluids for treatment of hyponatremia, other etiologies including SIADH should be kept in mind and workup should be started. Our case is one of the few cases reported in literature for SIADH associated with squamous cell carcinoma of pharynx.

## Figures and Tables

**Table 1:** Table with brief review of cases of SIADH secondary to pharyngeal squamous cell carcinoma.

References	Age (years) and gender	Location of tumor	Serum sodium (meq/L)
Okutomi et al. [22]	57, male	Floor of mouth	119
Thompson and Adlam [23]	71, male	Oral cavity	130
Danielides et al. [24]	67, male	Oral cavity	116
Kavanagh et al. [7]	57, male	Right nasopharynx	114
Yoo et al. [9]	77, male	Tonsillar mass	120
Krmar et al. [25]	8, male	Left pharynx	118
Suzuki et al. [26]	52, male	Hypopharynx	119
Zohar et al. [27]	62, male	Glottis and pharynx	126
